# Ferritin H subunit gene is specifically expressed in melanophore precursor-derived white pigment cells in which reflecting platelets are formed from stage II melanosomes in the periodic albino mutant of *Xenopus laevis*

**DOI:** 10.1007/s00441-015-2133-8

**Published:** 2015-02-26

**Authors:** Toshihiko Fukuzawa

**Affiliations:** Department of Biology, Keio University, Hiyoshi 4-1-1, Kohoku-ku, Yokohama, 223-8521 Japan

**Keywords:** Melanophore, Iridophore, Ferritin H subunit, Periodic albino, *Xenopus laevis*

## Abstract

“White pigment cells” are derived from melanophore precursors and contain both melanophore-specific and iridophore-specific pigment organelles. Whereas melanophores differentiate in the wild type regenerating tail, white pigment cells appear in the regenerating tail in the periodic albino mutant (*a* ^*p*^ */a* ^*p*^) of *Xenopus laevis*. The localization and density of white pigment cells in the mutant regenerating tail are similar to those of melanophores in the wild type regenerating tail. Here, white pigment cells in the mutant regenerating tail have been compared with melanophores in the wild type regenerating tail in the presence of phenylthiourea (PTU), which inhibits melanosome maturation in melanophores but does not affect reflecting platelet formation in white pigment cells. Ultrastructural analysis shows that reflecting platelet formation in white pigment cells is different from that in iridophores. Reflecting platelets in iridophores are formed from spherical vesicles with electron-dense material, whereas they are formed from stage II melanosomes characteristic of melanophore precursors in white pigment cells. Ultrastructural features of pigment organelles, except reflecting platelets, are similar between mutant melanophores and white pigment cells. In an attempt to identify specific genes in white pigment cells, a subtracted cDNA library enriched for mutant cDNAs has been prepared. Subtracted cDNA fragments have been cloned and selected by whole mount in situ hybridization. Among cDNA fragments examined so far, the ferritin H subunit gene is specifically expressed in white pigment cells, but not in melanophores. Pigment organellogenesis and specific gene expression in white pigment cells are also discussed.

## Introduction

Pigment cells are derived from neural crest cells in vertebrates (Bagnara and Hadley 1973; Hall and Hörstadius 1988; Le Douarin and Kalcheim 1999). A wide variety of pigment cells are known in poikilotherms, including melanophores, iridophores, leucophores, xanthophores, erythrophores, and cyanophores. Mammals have only one kind of pigment cell, a pigment cell type equivalent to melanophores (Bagnara 1998). Genetic mutations leading to visible phenotypes have led to a better understanding of pigment cells. In mammals, many genes regulating melanosome biogenesis and melanocyte development have been identified by using coat color mutants (Bennett and Lamoreux 2003; Jackson 1997; Raposo and Marks 2007; Schiaffino 2010). Zebrafish pigmentation mutations have also been used to analyze the genetic control of melanophores, xanthophores, and iridophores (Kelsh 2004; Kelsh et al. 1996, 2000, 2009; Parichy 2006; Parichy et al. 1999, 2000a, 2000b).

In poikilotherms, the black or brown color of melanophores is attributable to melanin in melanosomes, whereas the silver or gold color of iridophores is caused by reflecting platelets (Bagnara and Hadley 1973). The process and genes involved in melanosome biogenesis have been elucidated in mammalian melanocytes (Raposo and Marks 2007; Schiaffino 2010). In contrast, little is known about the molecular mechanism controlling reflecting platelet formation in iridophores, although several genes have been suggested to be involved in iridophore development (Lister et al. 2006, 2011; Lopes et al. 2008; Ng et al. 2009). Recently, cell lineage analyses in zebrafish have revealed that melanophores and iridophores develop from a common precursor whose fate is regulated by *foxd3* and *mitfa* (Curran et al. 2010). Furthermore, the characterization of the transcriptomes of iridophores and melanophores has identified genes whose expression is enriched in iridophores (Higdon et al. 2013). Another approach by using small molecule inhibitors has been applied to the study of pigmentation pathways in zebrafish chromatophores (Colanesi et al. 2012).

The periodic albino mutant (*a* ^*p*^ */a* ^*p*^) of *Xenopus laevis* is useful for studying the development of both melanophores and bright-colored pigment cells (Fukuzawa 2004, 2010; Fukuzawa and Ide 1986, 1987; Hoperskaya 1975, 1981; MacMillan 1979, 1981). This mutant was originally characterized by the absence of melanin in oocytes, the appearance of melanin in retinal pigment epithelium (RPE) and in skin melanophores at larval stages, and the depigmentation of both RPE and melanophores in metamorphosed animals (Hoperskaya 1975, 1981). Mutant melanophores contain abnormal melanosomes with granular internal structures (Fukuzawa and Ide 1986; Hoperskaya 1981; Seldenrijk et al. 1982). The depigmentation of mutant melanophores has been suggested to occur as follows: early stage melanosomes are accumulated, whereas mature melanosomes decrease in number with cell proliferation (Fukuzawa and Ide 1986). Iridophores (Fukuzawa 2006; MacMillan 1979; MacMillan and Gordon 1981) and xanthophores (Fukuzawa 2006) have been shown to be affected in the periodic albino mutant. The author has reported that unusual light-reflecting pigment cells, which show the characteristic features of both melanophores and iridophores, specifically appear in the periodic albino mutant (Fukuzawa 2004, 2010). Unusual light-reflecting pigment cells, which have been called “leucophore-like cells” (Fukuzawa 2004) and then renamed “white pigment cells” (Fukuzawa 2010), specifically appear in this mutant and are localized where melanophores normally differentiate in the wild type. White pigment cells are unique in (1) showing characteristics of melanophore precursors at various stages of development, (2) accumulating reflecting platelets characteristic of iridophores, and (3) exhibiting pigment dispersion in response to α-melanocyte stimulating hormone (α-MSH) in the same way that melanophores do (Fukuzawa 2010). By means of a tail-regenerating system, the study has shown that white pigment cells in the mutant regenerating tail are essentially similar to melanophores in the wild type regenerating tail with respect to their localization, number, and response to α-MSH. Therefore, white pigment cells in the mutant might arise from melanophore precursors and accumulate reflecting platelets characteristic of iridophores (Fukuzawa 2010).

The present study has been designed to elucidate the process of reflecting platelet formation and examine specific gene expression in white pigment cells in the periodic albino mutant. The *Xenopus* tadpole tail is best suited for the purpose of this study, because only white pigment cells are present in the posterior region of the mutant tadpole tail, whereas only melanophores localize in the same region of the wild type tadpole tail (Fukuzawa 2010). Using a tail-regenerating system, we have compared white pigment cells that differentiate in the mutant regenerating tail with differentiated melanophores in the wild type regenerating tail in the presence of phenylthiourea (PTU), an inhibitor of melanogenesis (Gross et al. 2002; Sims 1962).

To date, the formation of reflecting platelets in white pigment cells remains unclear. Ultrastructural studies of pigment cells have yielded important information concerning pigment organellogenesis (Bagnara et al. 1979a, 1979b; Bagnara 1998). Accordingly, we have observed pigment organelles by electron microscopy in iridophores, white pigment cells, and melanophores in culture.

In this study, we report that the ferritin H subunit mRNA is specifically expressed in white pigment cells but not in melanophores. This is the first report showing specific gene expression in white pigment cells in the periodic albino mutant. We also discuss the mechanism of pigment organellogenesis and specific gene expression in white pigment cells.

## Materials and methods

Wild type (+/+) and periodic albino mutant (*a* ^*p*^ */a* ^*p*^) *Xenopus laevis* were used. *Xenopus* eggs were obtained by gonadotropin stimulation, and developmental stages were determined according to Nieuwkoop and Faber (1967).

### Culture of iridophores and melanophores

Neural tubes of wild type and mutant embryos (stage 22) were used as the source of neural crest cells for differentiation into pigment cells (Fukuzawa and Ide 1988; Fukuzawa and Bagnara 1989; Fukuzawa 2006). The epidermis, somites, and notochord were removed from *Xenopus* embryos after 0.1 % collagenase treatment for 30 min. The cleaned neural tube was cultured in a sitting drop of 70 μl culture medium on a tissue culture dish (Falcon 3001; Becton Dickinson, Franklin Lakes, NJ, USA) at 25 °C. After 2 days, 2 ml medium was added to the culture. Subsequently, the medium was changed every 5 days. The culture medium consisted of 5 parts Leibovitz’s L-15 (Gibco, Grand Island, NY, USA), 3 parts Milli-Q ultrapure water (Millipore, Tokyo, Japan), and 2 parts fetal bovine serum (Gibco; Fukuzawa 2004). Melanophores and iridophores differentiated from neural crest cells under these culture conditions were as described previously (Fukuzawa 2006).

### Culture of white pigment cells from periodic albino mutant *Xenopus*

To isolate white pigment cells, tails of mutant tadpoles (stage 52) were utilized. Tadpole tails were cut and washed with sterile Steinberg’s balanced salt solution (BSS; Jones and Elsdale 1963). The tails were then chopped with scissors and dissociated with 0.3 % collagenase for 30 min. The resultant cell suspension was collected, washed, and inoculated onto a tissue culture dish (Falcon 3001; Becton Dickinson). White pigment cells attached to the culture dishes and were cultured at 25 °C in the medium described above for the culture of iridophores and melanophores.

### Tail regeneration experiments in presence or absence of PTU

Wild type and mutant tadpoles (stage 50) were utilized for tail regeneration experiments. The distal 50 % of the tail was amputated with a sharp razor blade in Steinberg’s BSS. The tadpoles were healed in 10 % Steinberg’s BSS and then reared in tap water containing either no PTU (control) or 0.5 mM PTU. The pigment cells that appeared in the regenerating tail were counted and compared between the wild type and mutant.

### Electron microscopy

Melanophores, iridophores, and white pigment cells were examined by electron microscopy to identify pigment organelles. Both intact and cultured cells were fixed in 2.5 % glutaraldehyde in 0.1 M cacodylate buffer (pH 7.2) for 60 min at 4 °C, post-fixed in 2 % O_S_O_4_ in the same buffer for 60 min at 4 °C, dehydrated through a graded series of ethanol, and embedded in epoxy resin. Ultrathin sections were stained with uranyl acetate and lead citrate and observed by means of a JEOL JEM-1010 electron microscope.

### Suppressive subtractive hybridization

Total RNA was extracted from tails of wild type and mutant tadpoles at stage 48/49 by using the RNeasy Mini Kit (Qiagen, Valencia, CA, USA), and poly(A)^+^ mRNA was isolated by using the Oligotex-dT30 <Super> mRNA Purification Kit (Takara Bio, Shiga, Japan). A subtracted cDNA library, enriched for mutant cDNAs, was prepared by using the PCR-Select cDNA Subtraction Kit (Clontech, Mountain View, CA, USA) according to the manufacturer’s protocols. Subtracted cDNA fragments were cloned into the pGEM-T Easy Vector (Promega, Madison, WI, USA) and selected by whole mount in situ hybridization (WISH).

### Whole mount in situ hybridization

Among cDNA fragments examined so far, the ferritin H subunit mRNA was found to be expressed in white pigment cells. RNA probes for in situ hybridization were generated from the cDNA fragment (486 bp) whose nucleotide sequence was 99 % identical to the ferritin H subunit gene of *Xenopus laevis*. The DDBJ accession number of the cDNA sequence is LC010236. Antisense and sense RNA probes were generated by using the DIG (digoxigenin) RNA Labeling Kit (Roche, Basel, Switzerland). Wild type and mutant tadpoles at stage 48 were used to examine the expression of the ferritin H subunit mRNA. WISH was performed as described (Sive et al. 2000) except that BM purple was used as a substrate, and RNase treatment was omitted. Specimens were bleached to remove melanin by using a bleaching solution (Mayor et al. 1995) either before hybridization or after BM purple staining.

## Results

### PTU inhibited melanosome maturation in melanophores but did not affect reflecting platelet formation in white pigment cells in regenerating tail

**Fig. 1:**
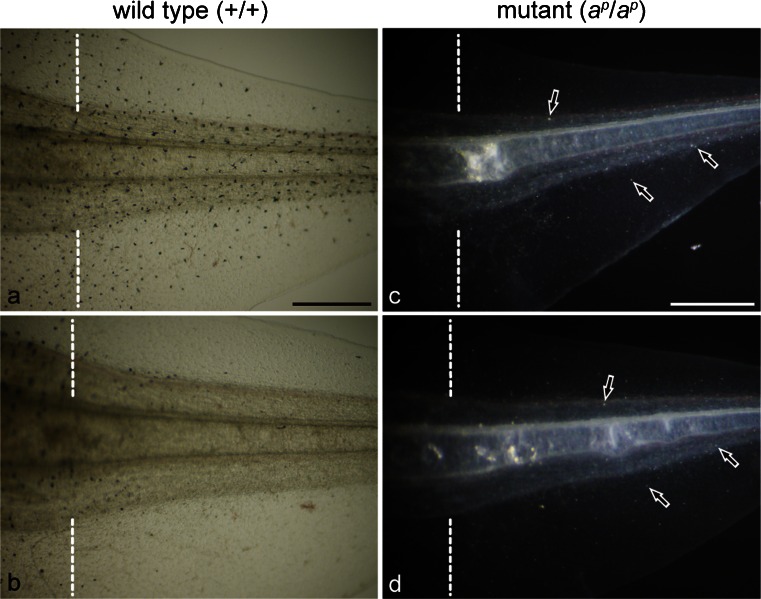
Expression of pigment cells in the 6-day regenerating tail in the absence (**a**, **c**) or presence (**b**, **d**) of phenylthiourea (PTU); amputated at stage 50. **a**, **b** Wild type regenerating tail observed under transmitted light. **c**, **d** Mutant regenerating tail observed under incident light. *Dashed lines* indicate amputation level. Many melanophores appeared in the wild type regenerating tail in the absence of PTU (**a**). However, few melanophores appeared in the wild type regenerating tail in the presence of PTU (**b**). In contrast, white pigment cells (*arrows*) appeared in the mutant regenerating tail in both the absence (**c**) and presence (**d**) of PTU. *Bar* 500 μm

When a tadpole tail was amputated at stage 50, a functionally competent new tail regenerated in both the wild type and the mutant regardless of the presence or absence of PTU (Fig. 1). Melanophores differentiated in the wild type regenerating tail in the absence of PTU (Fig. 1a), whereas white pigment cells differentiated in the mutant regenerating tail in the absence of PTU (Fig. 1c, arrows). The localization and density of white pigment cells in the mutant regenerating tail were similar to those of melanophores in the wild type regenerating tail as described previously (Fukuzawa 2010).

**Fig. 2:**
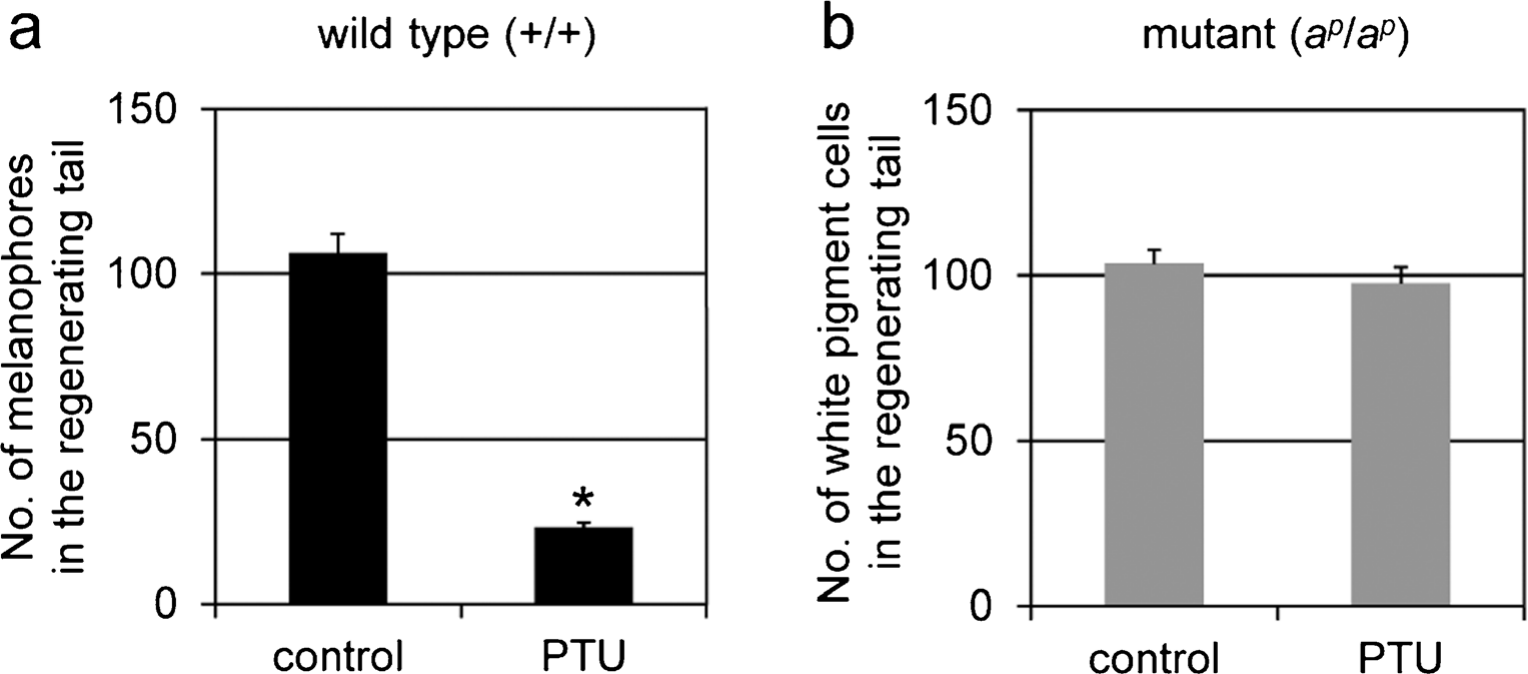
Effect of PTU on the number of differentiated pigment cells in the 5-day regenerating tail of the wild type (**a**) and the mutant (**b**); amputated at stage 50. **a** Melanophores were counted in the wild type regenerating tail in the absence (*control*; *n* = 24) or presence (*PTU*; *n* = 21) of PTU. **b** White pigment cells were counted in the mutant regenerating tail in the absence (*control*; *n* = 18) or presence (*PTU*; *n* = 15) of PTU. The number of melanophores in the wild type regenerating tail in the presence of PTU was statistically different from that in the absence of PTU (*t*-test, *P < 0.001). However, the number of white pigment cells in the mutant regenerating tail in the presence of PTU was not statistically different from that in the absence of PTU (*t*-test, *P* > 0.3)

In the presence of PTU, few melanophores appeared in the wild type regenerating tail (Fig. 1b). However, PTU did not affect the appearance of white pigment cells in the mutant regenerating tail (Fig. 1d, arrows). Figure 2a shows that the number of melanophores in the wild type regenerating tail in the presence of PTU was significantly lower than that in the absence of PTU (*P* < 0.001). In contrast, the number of white pigment cells in the mutant regenerating tail in the presence of PTU was not statistically different from that in the absence of PTU (*P* > 0.3; Fig. 2b).

**Fig. 3:**
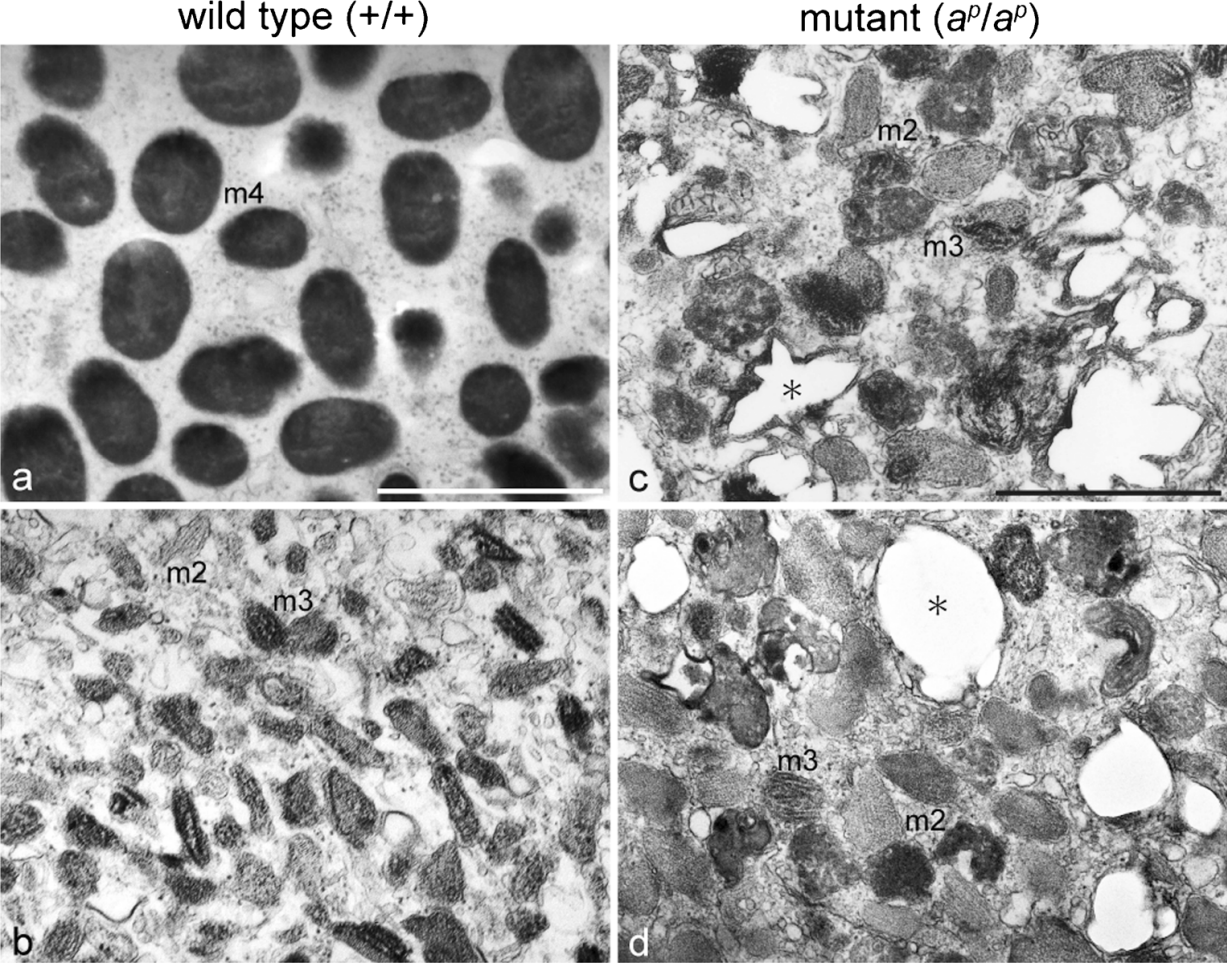
Ultrastructural characteristics of pigment cells that differentiated in the 18-day regenerating tail in the absence (**a**, **c**) or presence (**b**, **d**) of PTU (amputated at stage 50). **a**, **b** Ultrastructure of melanophores in the wild type regenerating tail. **c**, **d** Ultrastructure of white pigment cells in the mutant regenerating tail. Wild type melanophores in the absence of PTU (**a**) were filled with stage IV melanosomes (*m4*), whereas melanophores in the presence of PTU (**b**) contained stage II melanosomes (*m2*) and stage III melanosomes (*m3*). White pigment cells in both the absence (**c**) and presence (**d**) of PTU contained irregular-shaped reflecting platelets (*asterisks*), stage II melanosomes (*m2*), and stage III melanosomes (*m3*). The quantitative data on pigment organelles in the absence or presence of PTU are presented in Table 1 (wild type) and Table 2 (mutant). *Bar* 1 μm

Electron microscopic observation demonstrated that PTU inhibited melanosome maturation in melanophores in the wild type regenerating tail (Fig. 3, Table 1). Melanophores in the absence of PTU were filled with fully melanized stage IV melanosomes (Fig. 3a); however, melanophores in the presence of PTU contained stage II melanosomes and partially melanized stage III melanosomes (Fig. 3b). Table 1 shows that stage IV melanosomes decreased in number; however, stage I, II, and III melanosomes increased in the wild type regenerating tail melanophores in the presence of PTU (*P* < 0.001).

**Table 1:** Effect of phenylthiourea (*PTU*) on melanosome genesis in wild type melanophores

Treatment	Stage I + II^a^ / total	Stage III^a^ / total	Stage IV^a^ / total
Control^b^	0 %	0 %	100 %
PTU^c^	28.1 %*	69.8 %*	2.1 %*

*P < 0.001 (*χ* ^2^ test)

^a^Percentage of melanosomes at each stage among total melanosomes. Stage I and II melanosomes, partially melanized stage III melanosomes, and fully melanized stage IV melanosomes were according to the mammalian nomenclature of developing melanosomes (Raposo and Marks 2007)

^b^721 melanosomes were counted in 6 different photographs of melanophores in the 18-day wild type regenerating tails in the absence of PTU (see Fig. 3a)

^c^815 melanosomes were counted in 5 different photographs of melanophores in the 18-day wild type regenerating tails in the presence of PTU (see Fig. 3b)

White pigment cells in the mutant regenerating tail were characterized by the presence of both iridophore-specific and melanophore-specific pigment organelles. Regardless of the presence (Fig. 3d) or absence (Fig. 3c) of PTU, white pigment cells in the mutant regenerating tail contained irregular-shaped reflecting platelets, stage II melanosomes, and stage III melanosomes. The percentage of stage I and II melanosomes, stage III melanosomes, and reflecting platelets among total pigment organelles in the presence of PTU was not statistically different from that in the absence of PTU (Table 2). Reflecting platelet formation in white pigment cells was not affected by PTU. On the other hand, stage IV melanosomes were absent in white pigment cells in the presence of PTU, although few mature melanosomes were present in white pigment cells in the absence of PTU (Table 2). Therefore, melanosome maturation might also be inhibited by PTU in white pigment cells.

**Table 2:** Effect of PTU on pigment organellogenesis in mutant white pigment cells

Treatment	Stage I + II^a^ / total	Stage III^a^ / total	Stage IV^a^ / total	Reflecting platelets^b^ / total
Control^c^	30.8 %	46.1 %	1.7 %	21.4 %
PTU^d^	33.2 %	48.1 %	0 %*	18.7 %

* *P* < 0.001 (*χ* ^2^ test)

^a^Percentage of melanosomes at each stage among total pigment organelles

^b^Percentage of reflecting platelets among total pigment organelles

^c^1108 pigment organelles were counted in 10 different photographs of white pigment cells in the 18-day mutant regenerating tails in the absence of PTU (see Fig. 3c)

^d^989 pigment organelles were counted in 11 different photographs of melanophores in the 18-day mutant regenerating tails in the presence of PTU (see Fig. 3d)

### Ultrastructural features of pigment organelles, except reflecting platelets, were similar between mutant melanophores and white pigment cells

**Fig. 4:**
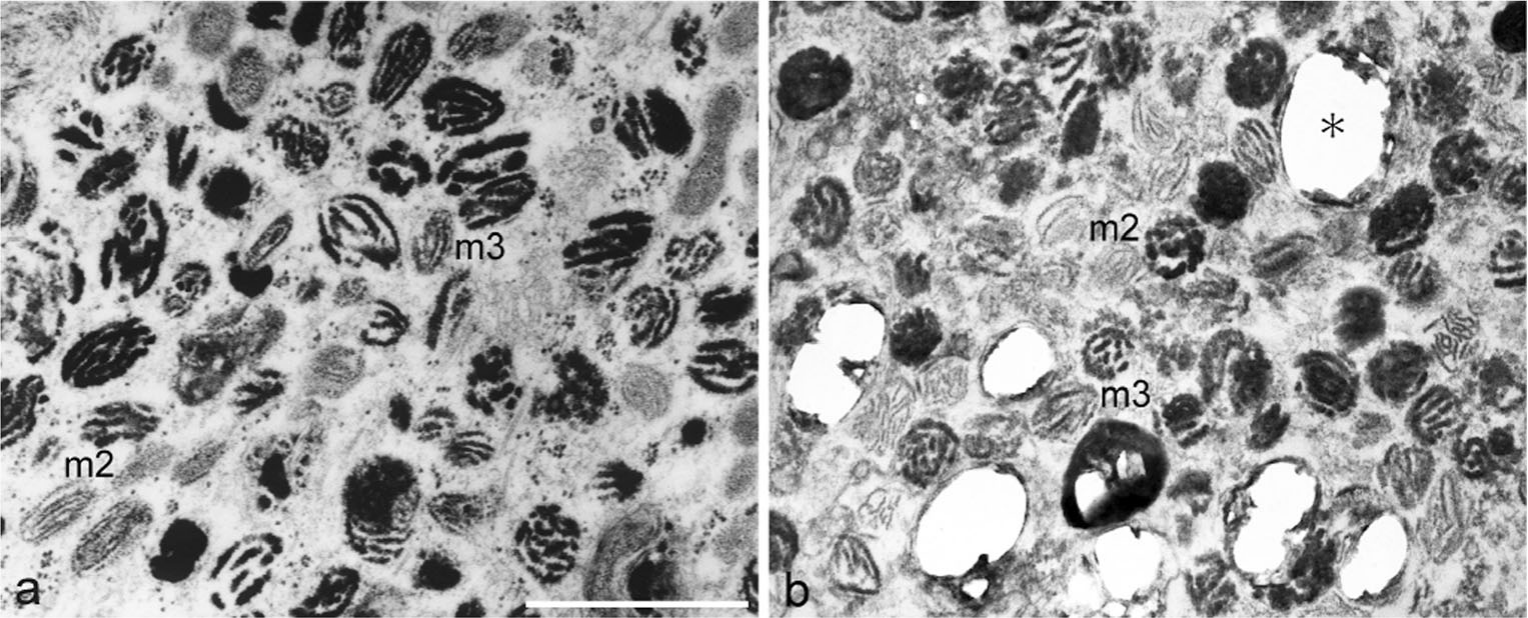
Ultrastructural comparison between mutant melanophores (**a**) and white pigment cells (**b**) in culture. Mutant melanophores in culture contained stage II melanosomes (*m2*), stage III melanosomes (*m3*), and few mature melanosomes. Cultured white pigment cells in the mutant contained stage II melanosomes (*m2*), stage III melanosomes (*m3*), and irregular-shaped reflecting platelets (*asterisk*). Few mature melanosomes were present in white pigment cells. *Bar* 1 μm

Melanophores differentiated from neural crest cells in vitro as described previously (Fukuzawa 2004). Cultured mutant melanophores contained stage II melanosomes, stage III melanosomes, and few mature melanosomes (Fig. 4a). Mutant melanophores were maintained for at least 2 months in culture. Transdifferentiation from mutant melanophores into white pigment cells did not occur at least under the present culture condition. Differentiated wild type melanophores were maintained without transdifferentiation in culture and contained many mature melanosomes (data not shown).

White pigment cells were isolated and cultured from mutant tadpole tails. Cultured white pigment cells contained stage II melanosomes, stage III melanosomes, and irregular-shaped reflecting platelets (Fig. 4b). Few mature melanosomes were present in cultured white pigment cells. These observations showed that ultrastructural features of pigment organelles, except reflecting platelets, were similar between mutant melanophores and white pigment cells.

### Process of reflecting platelet formation in white pigment cells was different from that in iridophores

**Fig. 5:**
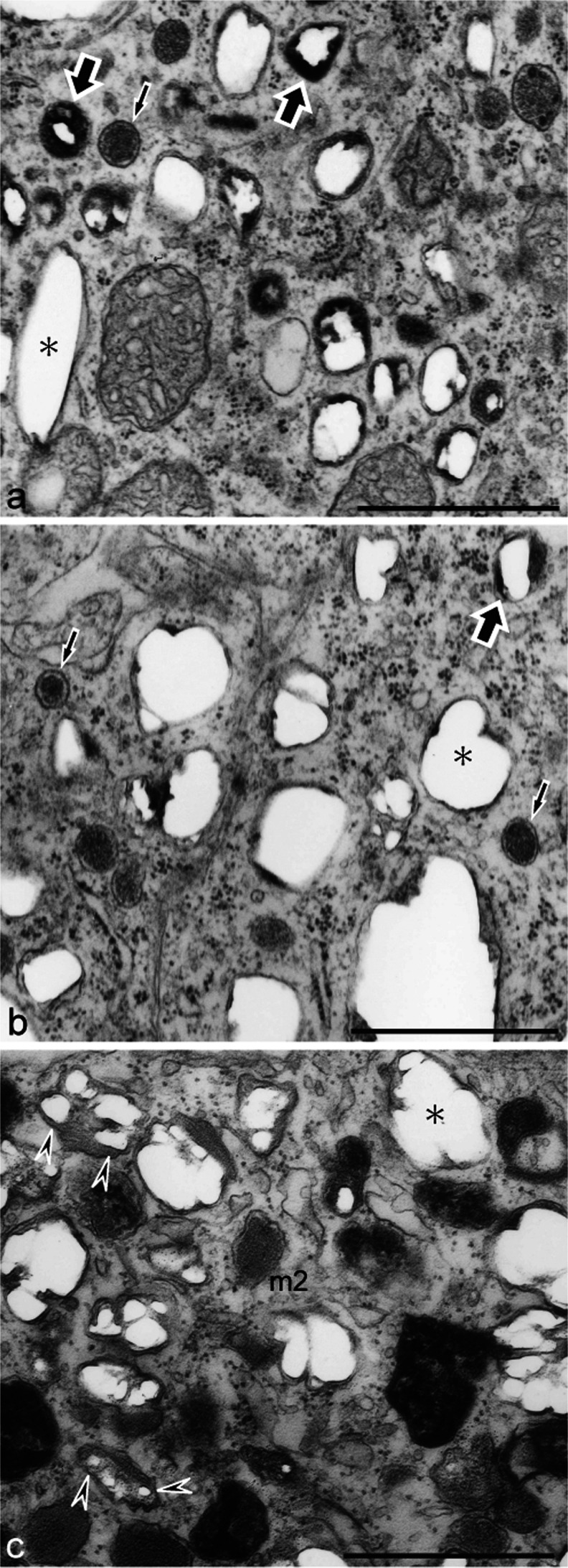
Reflecting platelet organellogenesis in iridophores and white pigment cells. **a**, **b** Ultrastructure of wild type (**a**) and mutant (**b**) iridophores that were allowed to differentiate in culture. **c** Ultrastructure of white pigment cells in the mutant regenerating tail. Spherical vesicles with electron-dense material (**a**, **b**, *small arrows*) were present in both wild type and mutant iridophores. Spherical vesicles subsequently accumulated crystals that were lost partially during fixation and thin-sectioning, leaving “partial holes” (*large arrows*) in the sections. Mature reflecting platelets were characterized by “empty holes” (*asterisk*). Reflecting platelets of wild type iridophores were rectangular (**a**, *asterisk*); however, those of mutant iridophores were irregular in size and shape (**b**, *asterisk*). White pigment cells in the mutant contained irregular-shaped reflecting platelets (**c**, *asterisk*) and stage II melanosomes with internal lamellar structures (*m2*). Note that reflecting platelets in white pigment cells were formed from stage II melanosomes (**c**, *arrowheads*), but not from spherical vesicles that were observed in iridophores (**b**). *Bar* 1 μm

The process of reflecting platelet formation in cultured iridophores was observed by electron microscopy in order to compare it with the process in white pigment cells. Differentiating iridophores in both the wild type and the mutant contained spherical vesicles with electron-dense material (Fig. 5a, b, small arrows). These vesicles subsequently accumulated crystals, which were partially lost during fixation and thin-sectioning, leaving “partial holes” in the sections (Fig. 5a, b, large arrows). Mature reflecting platelets, in which crystals grew to larger sizes, were characterized by “empty holes” (Fig. 5, b, asterisk) because almost all crystals were lost in the sections. Reflecting platelets of wild type iridophores were rectangular (Fig. 5a), whereas those of mutant iridophores were irregular in size and shape (Fig. 5b).

In addition to stage II melanosomes, white pigment cells in the mutant contained irregular-shaped reflecting platelets (Fig. 5c, asterisk). Among stage II melanosomes present in white pigment cells, some contained the same “partial holes” that were observed in reflecting platelets in iridophores (Fig. 5c, arrowheads). However, spherical vesicles that were observed in iridophores were absent in white pigment cells. These observations indicated that reflecting platelets in white pigment cells were formed from stage II melanosomes (Fig. 5c, arrowheads) but not from the spherical vesicles that were observed in iridophores (Fig. 5b).

### Ferritin H subunit mRNA was expressed in white pigment cells but not in melanophores

**Fig. 6:**
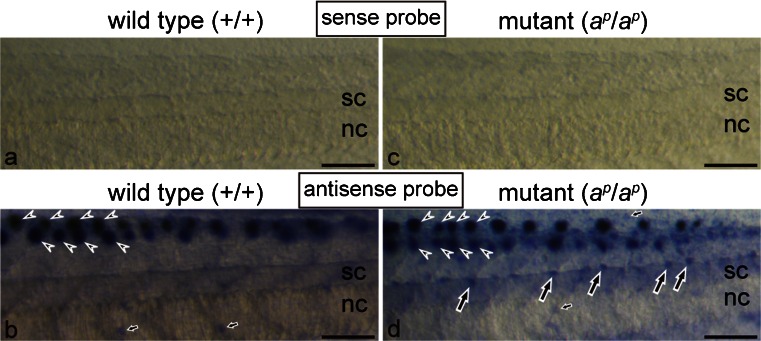
Spatial expression of the ferritin H subunit mRNA in the middle region of the tail in the wild type (**a**, **b**) and the mutant (**c**, **d**) at stage 48. Whole mount in situ hybridization (WISH) was performed by using sense (**a**, **c**) or antisense (**b**, **d**) digoxigenin (DIG)-labeled RNA probes. Tadpoles were bleached to remove melanin before hybridization in this experiment. With a sense probe of the ferritin H subunit mRNA, no staining was observed in the tail of both the wild type (**a**) and the mutant (**c**) in the negative control. Use of an antisense probe in WISH detected strong staining in the lateral lines (*arrowheads*) of both the wild type (**b**) and the mutant (**d**). In addition, specific expression of the ferritin H subunit mRNA was detected in white pigment cells (**d**, *large arrows*), which were present around the dorsal side of the spinal cord (*sc*) in the mutant (*nc* notochord). Although melanophores were present around the dorsal side of the spinal cord in the wild type, no staining was observed in melanophores (**b**). Note that staining was also detected in some epidermal cells (*small arrows*) in both the wild type (**b**) and the mutant (**d**). *Bar* 100 μm

WISH was performed in wild type and mutant tadpoles at stage 48 to examine the expression of the ferritin H subunit gene. Figure 6 shows the result of WISH in the middle region of the tail in which melanin was removed by bleaching before hybridization. In the negative control with a sense probe of the ferritin H subunit mRNA, no staining was observed in the tail of either the wild type (Fig. 6a) or the mutant (Fig. 6c). Hybridization with an antisense probe of the ferritin H subunit mRNA provided a clear hybridization signal (Fig. 6b, d). Strong staining was detected in the lateral lines of both the wild type and the mutant (Fig. 6b, d arrowheads). This result parallels the report of the ferritin H subunit gene being expressed in the accessory cells in the zebrafish lateral line (Behra et al. 2012). Some epidermal cells also expressed the ferritin H subunit mRNA in both the wild type and the mutant (Fig. 6b, d, small arrows). Specific expression of the ferritin H subunit mRNA was detected in white pigment cells, which were present around the dorsal side of the mutant spinal cord (Fig. 6d, large arrows). Although the localization of melanophores in the wild type was similar to that of white pigment cells in the mutant, no staining was observed in melanophores (Fig. 6b).

**Fig. 7:**
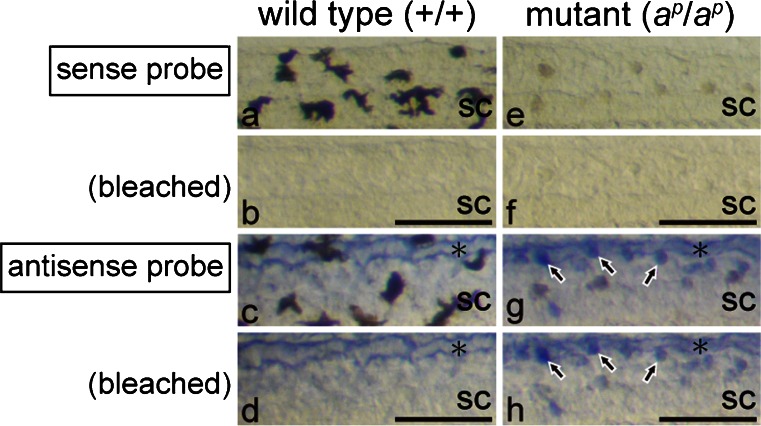
Expression of the ferritin H subunit mRNA in white pigment cells but not in melanophores. Photographs of the posterior region of the wild type tail (**a-d**) and the mutant tail (**e-h**) at stage 48. WISH was performed with sense (**a**, **b**, **e**, **f**) or antisense (**c**, **d**, **g**, **h**) DIG-labeled RNA probes (*sc* spinal cord). Tadpoles were bleached after BM purple staining. The same fields before (**a**, **c**, **e**, **g**) and after (**b**, **d**, **f**, **h**) bleaching are shown. In the negative control, with a sense probe of the ferritin H subunit mRNA, no staining was observed in the tail of the wild type (**a**, **b**) or the mutant (**e**, **f**). Before bleaching, dendritic black melanophores (**a**) were distinguished from punctate white pigment cells (**e**), which appeared brown under transmitted light. Melanin was bleached effectively in both melanophores (**b**) and white pigment cells (**f**). Staining with an antisense probe indicated that white pigment cells (**g**, **h**, *arrows*), but not melanophores (**c**, **d**), expressed the ferritin H subunit mRNA. Note that staining was also detected in the dorsal longitudinal anastomosing vessel (*asterisks*) in both the wild type (**c**, **d**) and the mutant (**g**, **h**). *Bar* 100 μm

To confirm the expression of the ferritin H subunit mRNA in white pigment cells, melanin was removed by bleaching after visualization of the hybridization signal with BM purple (Fig. 7). In the posterior region of the tail, melanophores had a dendritic shape and appeared black in the wild type (Fig. 7a), whereas white pigment cells had a punctate shape and appeared brown in the mutant under transmitted light (Fig. 7e). After the bleaching step, melanin disappeared in both melanophores (Fig. 7b) and white pigment cells (Fig. 7f). No staining was observed in the tail of either the wild type (Fig. 7a, b) or the mutant (Fig. 7e, f) by using a sense probe of the ferritin H subunit mRNA. Staining with an antisense probe clearly demonstrated that white pigment cells (Fig. 7g, h, arrows), but not melanophores (Fig. 7c, d), expressed the ferritin H subunit mRNA. Staining was also detected in the dorsal longitudinal anastomosing vessel in both the wild type (Fig. 7c, d, asterisk) and the mutant (Fig. 7g, h, asterisk). However, bleaching either before hybridization or after BM purple staining did not alter the staining pattern in the present experiment, except that melanin was removed.

**Fig. 8:**
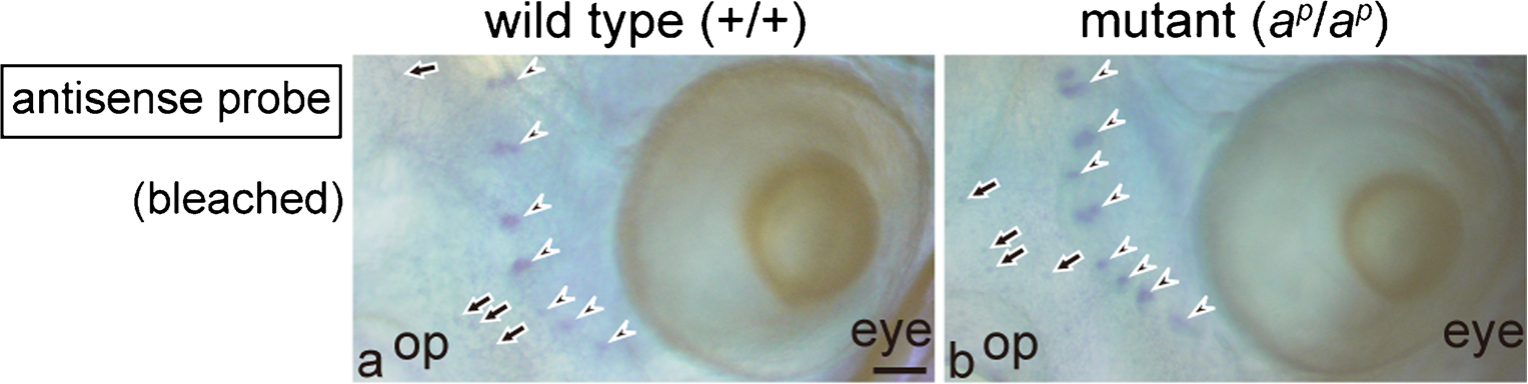
Ferritin H subunit mRNA is not expressed in the eye in the wild type (**a**) and the mutant (**b**) at stage 48. WISH was performed with sense (not shown) or antisense DIG-labeled RNA probes, and tadpoles were bleached after BM purple staining (*op* olfactory pit). No staining was observed in the eye in either the wild type (**a**) or the mutant (**b**). In contrast, expression of the ferritin H subunit mRNA was detected in the lateral lines (*arrowheads*) and some epidermal cells (*arrows*). *Bar* 100 μm

On the other hand, the ferritin H subunit mRNA was not expressed in the eye, including iridophores and RPE in both the wild type (Fig. 8a) and the mutant (Fig. 8b). In the head region, the ferritin H subunit mRNA was detected in the lateral lines (Fig. 8, arrowheads) and some epidermal cells (Fig. 8, arrows) as described in the tail (Fig. 6).

## Discussion

### Effect of PTU on melanophores and white pigment cells in regenerating tail

Previous reports have indicated that PTU inhibits the biosynthesis of melanin but does not influence the formation of early stage melanosomes (Eppig 1970; Pietzsch-Rohrschneider 1977). Because white pigment cells are derived from melanophore precursors and contain melanosomes in various stages of development, determination of whether PTU affects reflecting platelet formation in white pigment cells is of interest. Accordingly, the effect of PTU on melanophores and white pigment cells in the regenerating tail has been examined.

Few melanophores appeared in the wild type regenerating tail in the presence of PTU (Figs. 1, 2) because PTU inhibited melanosome maturation (Fig. 3; Table 1). The number of melanophores in the wild type regenerating tail in the presence of PTU was significantly lower than that in the absence of PTU (Fig. 2a). In the presence of PTU, the number of stage IV melanosomes decreased; however, the number of stage I, II and III melanosomes increased in melanophores in the wild type regenerating tail (Table 1). The number of melanophore precursors is probably unchanged; however, melanization is inhibited by PTU.

PTU did not affect the appearance of white pigment cells in the mutant regenerating tail (Figs. 1, 2). The number of white pigment cells in the presence of PTU was not statistically different from that in the absence of PTU (Fig. 2b). White pigment cells in the mutant regenerating tail contained irregular-shaped reflecting platelets and stage II and stage III melanosomes regardless of the presence or absence of PTU (Fig. 3). The percentage of stage I and II melanosomes, stage III melanosomes, and reflecting platelets among total pigment organelles in the presence of PTU was not statistically different from that in the absence of PTU (Table 2). Therefore, these data showed that reflecting platelet formation in white pigment cells was not affected by PTU. On the other hand, stage IV melanosomes were absent in white pigment cells in the presence of PTU, although few mature melanosomes were observed in white pigment cells in the absence of PTU (Table 2). This result can be interpreted as the inhibition of melanosome maturation by PTU in both melanophores and white pigment cells in the regenerating tail.

### Difference between melanophores and white pigment cells in periodic albino mutant

**Fig. 9:**
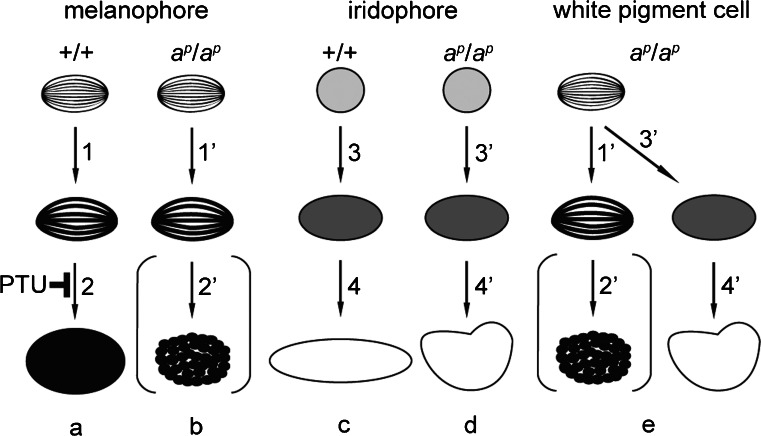
Representation of pigment organellogenesis in melanophores (**a**, **b**), iridophores (**c**, **d**), and white pigment cells (**e**). **a**, **b** Melanosome formation in wild type (**a**) and mutant (**b**) melanophores. **c**, **d** Reflecting platelet formation in wild type (**c**) and mutant (**d**) iridophores. **e** Pigment organellogenesis in white pigment cells in the mutant. In wild type melanophores, melanin deposition occurs in stage II melanosomes to form partially melanized stage III melanosomes (**a**, *1*) and then fully melanized stage IV melanosomes are formed (**a**, *2*). Melanin is also deposited in stage II melanosomes to form stage III melanosomes (**b**, *1’*) in mutant melanophores; however, few melanosomes become fully melanized (**b**, *2’*). PTU inhibits melanosome maturation from stage III melanosomes to stage IV melanosomes in melanophores. In wild type iridophores, spherical vesicles with electron-dense material accumulate crystals (**c**, *3*), which grow larger and exhibit rectangular reflecting platelets (**c**, *4*). Crystals are also accumulated in spherical vesicles with electron-dense material in mutant iridophores (**d**, *3’*); however, reflecting platelets become irregular in shape (**d**, *4’*). In white pigment cells, melanosome formation (**e**, *1’*, *2’*) occurs in the same manner as described in mutant melanophores (**b**, *1’*, *2’*). In addition, some stage II melanosomes accumulate crystals in white pigment cells (**e**, *3’*), and irregular-shaped reflecting platelets are formed (**e**, *4’*)

Mutant melanophores were maintained without transdifferentiation in culture (Fig. 4a). Cultured mutant melanophores were filled with stage II and stage III melanosomes (Fig. 4a). However, few mature melanosomes were present in mutant melanophores in culture. In contrast, many mature melanosomes were contained in the wild type melanophores in culture as described previously (Fukuzawa and Ide 1986). Representations of melanosome formation in wild type and mutant melanophores are shown in Fig. 9a, b, respectively. PTU treatment partially phenocopies melanophores in the periodic albino mutant (Fig. 9a, b).

White pigment cells could be isolated from mutant tadpole tails and cultured in vitro. Cultured white pigment cells showed the same characteristic features as white pigment cells in vivo. Transdifferentiation from melanophores into white pigment cells did not occur under the present culture condition. Early differentiating melanophores and late appearing white pigment cells might be derived from different melanophore lineages in the periodic albino mutant. This idea is supported by a finding that two distinct melanophore lineages exist in zebrafish (Hultman et al. 2009; Hultman and Johnson 2010). Recently, melanophores have been demonstrated to be derived from either pluripotent neural crest cells expressing *foxd3* or bipotent pigment precursors expressing *mitfa* in zebrafish (Curran et al. 2010). Further studies are needed to clarify the lineage of melanophores in the periodic albino mutant.

### Reflecting platelet formation in iridophores and white pigment cells

Based upon the observation of mosaic pigment cells, a hypothetical model has been proposed with pigment cells derived from a stem cell that contains a primordial organelle (Bagnara et al. 1979a, 1979b). Although the process of melanosome biogenesis has been analyzed genetically, little is known about the molecular mechanism of reflecting platelet formation.

White pigment cells are different from iridophores in size, shape, blue-light-induced fluorescence, and their response to α-MSH, although reflecting platelets are contained in both of them (Fukuzawa 2010). The difference between white pigment cells and iridophores is thought to reflect their different origin, namely, iridophores are derived from iridoblasts, whereas white pigment cells arise from melanophore precursors (Fukuzawa 2010).

The process of reflecting platelet formation in mutant iridophores was the same as that in wild type iridophores (Fig. 5a, b), although the size and shape of reflecting platelets were different between wild type and mutant iridophores as described previously (Fukuzawa 2006, 2010). In both wild type and mutant iridophores, spherical vesicles with electron-dense material (Fig. 5a, b, small arrows) accumulated crystals and formed reflecting platelets, which were characterized by “partial holes” (Fig. 5a, b, large arrows) or “empty holes” (Fig. 5a, b, asterisk), because crystals were lost during fixation and thin-sectioning (Bagnara et al. 1979b; Morrison and Frost-Mason 1991). Indeed, mature reflecting platelets have been characterized by “empty holes” in various species (Matsuno and Iga 1989; Morrison and Frost-Mason 1991; Nagaishi and Oshima 1992; Rohrlich and Porter 1972; Taylor 1969). The process of reflecting platelet formation observed in *Xenopus* iridophores in the present study is consistent with the previous observation in lizard skin iridophores (Morrison and Frost-Mason 1991). Representations of reflecting platelet formation in wild type and mutant iridophores are shown in Fig. 9c, d, respectively.

The present observations demonstrated that the process of reflecting platelet formation in white pigment cells was different from that in iridophores (Fig. 5). Reflecting platelets in white pigment cells were formed from stage II melanosomes (Fig. 5c, arrowheads) and not from the spherical vesicles that were observed in iridophores (Fig. 5b). A representation of reflecting platelet formation in white pigment cells is shown in Fig. 9e.

At this time, the genes involved in reflecting platelet formation from stage II melanosomes are not known. A number of genes involved in purine synthesis have been reported to be upregulated in iridophores (Higdon et al. 2013). We cannot exclude that the upregulation of enzymes in the purine synthesis pathway stimulates reflecting platelet genesis in stage II melanosomes in white pigment cells.

Several factors are known to be important for iridophore development, including *ltk*, *endrb1*, and *pnp4a* (Curran et al. 2010; Lopes et al. 2008; Parichy et al. 2000a). Recently, an interesting finding has been reported that *gpnmb*, which is suggested to act as a plasma membrane protein and a component of the melanosome, is highly expressed in iridophores (Higdon et al. 2013). A similar function might therefore exist in melanosome biogenesis and reflecting platelet formation.

### Factors that affect pigment organellogenesis

The regulation of pH has been shown to be important in melanosome formation (Ancans et al. 2001; Raposo and Marks 2007; Schiaffino 2010; Smith et al. 2004). Whereas a low pH is required to form stage I melanosomes, an increase in pH is required for melanogenesis and melanosome maturation, because the optimal pH of tyrosinase is near neutral (Ancans et al. 2001; Dooly et al. 2012; Smith et al. 2004). In zebrafish, *slc45a2*, V-ATPase, and *slc24a5* have been shown to control pH and ionic homeostasis in melanosome biogenesis (Dooly et al. 2012). On the other hand, the iron ion has been recently reported to affect melanosomes in RPE (Wolkow et al. 2011). In aceruloplasminemia and age-related macular degeneration (AMD), depigmentation of RPE is correlated with increased levels of iron, because melanosomes in RPE can be degraded via iron-mediated reactive oxygen species production (Wolkow et al. 2011).

The iron storage protein, ferritin, plays an important role in iron metabolism and performs a protective antioxidant function in a wide variety of cell types (Arosio et al. 2009; Harrison and Arosio 1996). Iron and ferritin have been suggested to be involved in melanosome degradation in RPE (Wolkow et al. 2011). Upregulation of the ferritin gene has been demonstrated in melanocytes and keratinocytes in the presence of skin lightening agents (Gruber and Holtz 2013). Experiments with a human RPE cell line have shown that melanosomes are competent to bind iron, and that ferritin levels are increased by increasing levels of iron (Kaczara et al. 2012). Therefore, we propose that iron and ferritin are involved in the depigmentation of both RPE and melanophores in the periodic albino mutant.

In the *Xenopus* tadpole, the ferritin H subunit mRNA is expressed in the lateral lines and some epidermal cells (Figs. 6, 8). Expression of the ferritin H gene has also been reported in neural tissues in *Xenopus laevis* neurula (Shin et al. 2005). A surprising finding in the present study is that white pigment cells, but not melanophores, express the ferritin H subunit gene (Figs. 6, 7). However, the ferritin H subunit gene is not expressed in the eye, including iridophores and RPE (Fig. 8).

We are tempted to speculate that ferritin, together with pH and ionic conditions, is involved in unusual pigment organellogenesis in white pigment cells. Further studies are necessary to understand the mechanism of pigment organellogenesis, including that of melanosomes and of reflecting platelets.
